# Supplementary material for: Reconciling Mining with the Conservation of Cave Biodiversity: A Quantitative Baseline to Help Establish Conservation Priorities
Source: PLoS One. 2016 Dec 20;11(12):e0168348. doi: 10.1371/journal.pone.0168348 (PMC5173368; doi:10.1371/journal.pone.0168348)
Supplement: S1 Dataset — (ZIP) [file pone.0168348.s002.zip › Taxa/Serra Norte/SN_2013/N5S/Lista N5S-56.pdf]

CAVIDADE N5S-0056

Classe	Ordem	Fam/Outros	Gên/Outros	Espécie	S-U	U-S	Zon.
Annelida	Oligochaeta			jovem		1	P
Arachnida	Amblypygi	Phrynidae	*Heterophrynus*	*longicornis*	4		P
Arachnida	Araneae	Caponiidae		jovem	X		P
Arachnida	Araneae	Corinnidae		jovem		2	P
Arachnida	Araneae	Ctenidae		jovem	1		E
Arachnida	Araneae	Dipluridae		sp.2	1		E
Arachnida	Araneae	Drymusidae		jovem	X	X	E/P
Arachnida	Araneae	Ochyroceratidae		jovem	X		P
Arachnida	Araneae	Oonopidae	aff. *Triaeris*	sp.2	X		E
Arachnida	Araneae	Oonopidae	aff. *Xyccarph*	sp.2		X	P
Arachnida	Araneae	Pholcidae	*Mesabolivar*	*aurantiacus*		X	P
Arachnida	Araneae	Salticidae		jovem	X		E
Arachnida	Araneae	Scytodidae	*Scytodes*	jovem	1		E
Arachnida	Araneae	Theridiosomatidae	*Plato*	sp.1	X	X	E/P
Arachnida	Opiliones	Cosmetidae	*Roquettea*	*singularis*	2		E
Arachnida	Opiliones	Escadabiidae		sp.1	X		P
Arachnida	Opiliones	Escadabiidae		sp.2	X		P
Arachnida	Opiliones	Gonyleptidae	Tricommatinae	sp.1	X		E
Arachnida	Opiliones	Manaosbiidae	*Saramacia*	sp.1	1		E
Arachnida	Opiliones	Stygnidae	*Protimesius*	*gracilis*	2		E
Arachnida	Palpigradi	Eukoeneniidae	*Eukoenenia*	sp.1		X	E
Arachnida	Pseudoscorpiones	Bochicidae		sp.2		X	P
Arachnida	Pseudoscorpiones	Chthoniidae	*Pseudochthonius*	sp.1	X	X	E
Chilopoda	Scutigeromorpha	Pselliodidae	*Sphendononema*	*guildingii*		2	E/P
Entognatha	Collembola	Paronellidae		sp.1		X	P
Entognatha	Collembola	Paronellidae		sp.4	X		P
Entognatha	Collembola	Paronellidae		sp.6	X		E
Entognatha	Collembola	Paronellidae		sp.7		X	E
Entognatha	Diplura	Campodeidae		sp.1	X	X	P
Insecta	Blattodea			jovem	2		E
Insecta	Coleoptera			jovem	X	X	E/P
Insecta	Diptera	Psychodidae	Phlebotominae	sp.		X	P
Insecta	Diptera	Tipulidae	Tipulinae	sp.		X	E
Insecta	Heteroptera	Reduviidae	Emesinae	sp.	1		P
Insecta	Heteroptera	Reduviidae	Reduviinae	sp.	3		E
Insecta	Homoptera	Cixiidae		sp.4		X	E
Insecta	Hymenoptera	Formicidae	*Crematogaster*	sp.1	X		E
Insecta	Hymenoptera	Formicidae	*Paratrechina*	sp.1	X	X	E/P
Insecta	Hymenoptera	Formicidae	*Paratrechina*	sp.2		X	P
Insecta	Hymenoptera	Formicidae	*Pheidole*	sp.3	X	X	P
Insecta	Hymenoptera	Formicidae	*Solenopsis*	sp.1		X	P
Insecta	Hymenoptera	Formicidae	*Trachymyrmex*	sp.1	X	X	P
Insecta	Isoptera	Termitidae	*Embiratermes*	sp.		X	E
Insecta	Isoptera	Termitidae	*Nasutitermes*	sp.	X		E
Insecta	Isoptera	Termitidae	*Subulitermes*	sp.	X		E
Insecta	Lepidoptera	Tineoidea		sp.1	X	X	E
Insecta	Orthoptera	Phalangopsidae	*Paraclodes*	jovem	4	2	E/P
Insecta	Orthoptera	Phalangopsidae	*Phalangopsis*	jovem	1	14	P
Insecta	Thysanura	Nicoletiidae		sp.1	X	X	P

Gastropoda	Pulmonata	Subulinidae		sp.4		X	P
Malacostraca	Isopoda	Armadillidae		sp.1		X	P
Malacostraca	Isopoda	Philosciidae		sp.1	X	X	P
Symphyla		Scutigerellidae		sp.		X	E/P
Amphibia	Anura	Strabomantidae	*Pristimantis*	cf. *fenestratus*	1		E
